# Design of a dimeric‐nanobody specific for Aß_1‐42_ oligomers: the Dimeric‐DesAb‐O

**DOI:** 10.1002/alz.089585

**Published:** 2025-01-09

**Authors:** Liliana Napolitano, Devkee Vadukul, Fabrizio Chiti, Cristina Cecchi, Roberta Cascella, Francesco Aprile

**Affiliations:** ^1^ Università degli studi di Firenze, Firenze Italy; ^2^ Imperial College London, London United Kingdom

## Abstract

**Background:**

Small, soluble oligomers, rather than mature fibrils, are the major neurotoxic agents in Alzheimer’s disease (AD). In the last few years, Aprile and co‐workers designed and purified a single‐domain antibody (sdAb), called DesAb‐O, with high specificity for Aβ_1‐42_ oligomeric conformers. Recently, Cascella and co‐workers showed that DesAb‐O can selectively detect synthetic Aβ_1‐42_ oligomers both in vitro and in cultured cells, neutralizing their associated neuronal dysfunction. DesAb‐O can also identify Aβ_1‐42_ oligomers in the cerebrospinal fluid (CSF) of AD patients, with respect to healthy individuals, preventing cell dysfunction induced by the administration of CSFs to neuronal cells. Given the extraodinary potentialities of this nanobody, we design a dimeric‐structure of DesAb‐O, with the aim to increase its avidity and affinity for toxic Aβ_1‐42_ oligomers.

**Method:**

We designed the dimeric‐DesAb‐O structure by linking two DesAb‐O monomeric domains with a flexible linker region (GGGGS)_3._ Once expressed and purified the protein, we characterised its molecular weight by mass spectrometry and its secondary structure by Circular Dichroism. Then, we performed an aggregation assay to monitor its ability to interfere with the Aβ_1‐42_ aggregation process and a Real‐Time based ELISA assay to study its binding for Aβ_1‐42_ oligomers.

**Result:**

The Dimeric‐DesAb‐O is able to interfere with the Aβ_1‐42_ aggregation process to a greater extent than DesAb‐O. Furthermore, the dimeric structure of DesAb‐O showed a higher specificity and affinity for Aβ_1‐42_ oligomers compared to DesAb‐O.

**Conclusion:**

The Dimeric‐DesAb‐O appears to be a promising tool for the future development of sdAbs‐based immunodiagnostic tests for the early diagnosis of AD.

